# The detection of gastrointestinal parasites in owned and shelter dogs in Cebu, Philippines

**DOI:** 10.14202/vetworld.2019.372-376

**Published:** 2019-03-07

**Authors:** Marysia Frances M. Urgel, Rochelle Haidee D. Ybañez, Adrian P. Ybañez

**Affiliations:** 1 College of Science, University of the Philippines Cebu, Gorordo Avenue, Lahug, Cebu City 6000, Philippines; 2 National Research Center for Protozoan Diseases, Obihiro University of Agriculture and Veterinary Medicine, Obihiro City, Hokkaido, Japan; 3 College of Veterinary Medicine at Barili Campus and Center for Vector-borne and Protozoan Diseases at Main Campus, Cebu Technological University, Cebu City 6000, Philippines

**Keywords:** Cebu, dogs, gastrointestinal parasites

## Abstract

**Background:**

Gastrointestinal tract (GIT) parasites affect the health of dogs and may also be zoonotic. The prevalence of these parasites has been well studied in several countries, but reports in the Philippines have been limited.

**Aim:**

This study generally aimed to detect the presence of common GIT parasites in owned and shelter dogs in Cebu, Philippines.

**Materials and Methods:**

A total of 200 fecal samples (130 from owned dogs and 70 from shelter dogs) were collected. Profiles of owned dogs and their owners were obtained. Fecalysis was performed using three methods: Direct smear, sedimentation, and flotation techniques.

**Results:**

Majority of the sampled dogs were 5 years old and below that (79.2%), male (64.6%) and of pure breed (53.1%). Among the most common parasites detected were *Ancylostoma*, *Trichuris* and *Toxocara* spp. Statistical analyses revealed a significant association between the presence of parasites and the body score of the dogs (p=0.000), the deworming status (p=0.000), and the rearing practice (contact with other dogs, p=0.000, where it spends its time (p=0.000), plays in the grass (p=0.050), where it defecates (p=0.014), contact with other animals (p=0.000).

**Conclusion:**

GIT parasites were detected in owned and shelter dogs in Cebu, Philippines. The results of this study can serve as baseline information about the canine parasitic fauna in the Philippines.

## Introduction

Companion animals live closely with humans. In some countries, the majority of households may own companion animals [1]. A mutual relationship between owners and their companion animal exist - humans provide shelter, food, and care while the companion animal contributes to the overall well-being of their owners [2]. Studies have shown that people who own companion animals have better health conditions than those who do not have any [3]. Among the most popular companion animal are the dogs, which may also come from shelter homes.

While a dog may contribute to the well-being of their owners, it can also be a host to different endoparasites. Some of these parasites are also detrimental to the owners as it can serve as carriers for different zoonotic diseases [4-5]. Dogs with parasite infestations show a variety of symptoms depending on the type of parasite and the density. The most common symptoms include intestinal disorder, anorexia, weight loss, anemia, and dehydration. Severe cases could be fatal when not immediately treated [6].

In different countries, the prevalence of endoparasites in dogs ranges from 5% to 70% [7-10]. These studies showed that among the most common canine intestinal parasites are the *Trichuris* spp*., Toxocara* spp*., Ancylostoma* spp., an*d Cystoisospora* spp. Having information on the presence of the common gastrointestinal tract (GIT) parasites in the area is essential for prevention measures and the diagnosis and treatment approach of local veterinarians. In Cebu, Philippines, there is no current study that evaluates the presence of GIT parasites in dogs. Hence, the present study aimed to determine the prevalence of GIT parasites in household and shelter dogs using different fecalysis methods.

## Materials and Methods

### Ethical approval

The procedures performed in this study were guided by the principles of animal welfare, Animal Welfare Act of the Philippines (RA 8485) and Administrative Order No. 45 of the Bureau of the Animal Industry of the Philippines.

### Samples and research area

A total of 200 dog stool samples (130 individual stool samples from different households and 70 stool pooled samples) from different shelter homes in Cebu, Philippines, were analyzed. Stool samples were pooled together into one in cages or kennels containing three or more dogs since it was difficult to ascertain in which dogs owned the samples. Profile of sampled dogs from the household, including age, sex, breed, anthelmintic usage (last treated, <12 months or >12 months ago or was never treated) [6], and selected rearing information practices, were obtained (Table-1). Fecalysis was performed at the Biology Laboratory of the University of the Philippines Cebu, Lahug, Cebu City, Philippines.

**Table-1:** Profile of household dogs and its selected rearing practices in Cebu, Philippines (n=130).

Parameter	Frequency (%)
Age	
<1 year old	26 (20.0)
>1-5 years old	77 (59.2)
>5-7 years old	12 (9.2)
>7 years old	15 (11.5)
Sex	
Male	84 (64.6)
Female	46 (35.4)
Breed	
Pure	69 (53.1)
Mixed	61 (46.9)
Body score	
Below normal	61 (46.9)
Ideal	62 (47.7)
Overweight	7 (5.4)
Dewormer given	
Yes	87 (66.9)
No	43 (33.1)
Present medical condition	
No apparent clinical signs	124 (95.4)
Pregnant	1 (0.8)
Distemper	1 (0.8)
Allergies	2 (1.5)
Mammary tumor	2 (1.5)
Previous medical condition	
None	117 (90)
Ehrlichia	2 (1.5)
Parvovirus	2 (1.5)
Anemia	1 (0.8)
Diarrhea	2 (1.5)
Lice	1 (0.8)
Mange	2 (1.5)
Allergies	2 (1.5)
Diarrhea, vomiting, lice	1 (0.8)
Symptoms during fecal collection	
None	125 (96.2)
Lethargy	3 (2.3)
Diarrhea	2 (1.5)
Contact with dogs belonging to other households	
Never	28 (21.5)
Rarely	65 (50)
Once a week	2 (1.5)
2-3 times a week	8 (6.2)
4-5 times a week	3 (2.3)
Daily	24 (18.5)
Place where it spends most of the time	
All the time outside	16 (12.3)
Mostly outside	24 (18.5)
Half outside, half inside	20 (15.4)
Mostly inside the house	43 (33.1)
Always inside the house	22 (16.9)
Unsure	5 (3.8)
Playing with grass	
Never	34 (26.2)
Rarely	60 (46.2)
Always	36 (27.7)
Place of defecation	
Litter Tray	2 (1.5)
Kennel/Cage	10 (7.7)
Street/Concrete	39 (30)
Grass/Lawn	45 (34.6)
Soil/Sand/Dirt	30 (23.1)
Grass and street	2 (1.5)
Others	2 (1.5)
Contact with other animals	
Never	34 (26.2)
Rarely	75 (57.7)
Daily	21 (16.2)

### Sampling procedure and fecalysis

Fecal sampling was conducted from February to May 2017. Feces were scooped using individual sticks and placed in properly labeled containers. After collection, samples were immediately transported to the laboratory under low temperature. On reaching the laboratory, samples were refrigerated awaiting analysis. Three fecalysis methods were performed as previously described: Direct fecal smear [11], sedimentation (Royal Veterinary College: Food and Agriculture Organisation Guide to Veterinary Diagnostic Parasitology), and floatation using passive technique and sugar [12]. In cases where samples could not be analyzed immediately, samples were mixed with 10% formalin and refrigerated at 4-8°C until further examination [13].

### Data processing and analysis

Gathered data were manually encoded in Microsoft Excel and imported to statistical software (IBM SPSS^®^, USA). Descriptive statistics were employed. Chi-square test was used for categorical variables (sex, gender, breed, and use of anthelmintics) and Mann–Whitney U-test was used for the age category. The significance level was set at 5%.

## Results and Discussion

Most of the owned dogs were male and belonged to the age category of above 1-5 years old (59.2%). The average age of the dogs that participated in the study is 3.1 years old. Majority of the owned dogs have an ideal body score and have been treated for GIT parasites. Most of the dewormed dogs were given a deworming treatment about 4-6 months ago, but the majority of the owners were unsure of the type of deworming treatment given to their dog. Most of the owned dogs stayed inside the house. Almost all of the owned dogs did not have any present medical conditions, previous medical conditions, or symptoms observed during fecal collection. Since most owned dogs stay inside the house, it rarely had contact with dogs belonging to other households.

Several GIT parasites were detected (Table-2). *Ancylostoma* spp. was found highest (38%) similar to other studies [6,10,14] that reported even >90% prevalence [14,15]. Transmission of this parasite can occur through penetration of skin at hair follicles or sweat glands or through direct ingestion *Ancylostoma caninum* [16,17].

**Table-2:** GIT parasites detected in Cebu, Philippines (n=200).

Parasite	House Dogs (n=130)	Shelter Dogs (n=70)	Total (n=200)
		
	Frequency (%)	Frequency (%)	Frequency (%)
*Ancylostoma* spp.	36 (27.7)	40 (57.1)	76 (38)
*Toxocara* spp.	12 (9.2)	11 (15.7)	23 (11.5)
*Trichuris* spp.	11 (8.5)	14 (20)	25 (12.5)
*Hammondia* spp.	0	3 (4.3)	3 (1.50
*Taenia* spp.	5 (3.9)	1 (1.4)	6 (3)
*Cystoisospora* spp.	1 (0.8)	14 (20)	16 (8)

GIT=Gastrointestinal tract

Eggs of *Trichuris* spp. were detected in 12.5% of the fecal samples. *Trichuris* spp. are considered to be soil-transmitted helminths [18,19]. In Thailand, the leading cause of helminthic infections included *Trichuris vulpis* (16%) [19].

*Toxocara* spp. was found in 11.5% of the fecal samples. These are roundworm eggs which can develop further into the larval stage. After leaving the definitive host, it grows and develops before becoming infectious. *Toxocara canis* can be transmitted to humans through the accidental ingestion of its eggs. *Toxocara* spp. was one of the more frequent parasites detected in dogs in several countries [6-10].

*Cystoisospora* spp. and *Hammondia* spp. were respectfully found in 8% and 1.5% of the fecal samples. Similar lower detection of *Cystoisospora* spp. (1.2%) was also reported in Japan [20]. *Cystoisospora* and *Hammondia* belong to the class *Conoidasida*, which infect the GIT of animals [21].

*Taenia* spp. was detected in 1.5% of dogs. The presence of *Taenia saginata* and *Taenia solium* has been reported in humans and food animals in a nearby island of Cebu, Philippines (Leyte) [22]. It was suggested in the aforementioned report that the infected humans may have acquired it by eating not well-cooked meat from food animals. Hence, it is possible that the five dogs that were positive in this study may have also eaten not well-cooked meat. *Taenia* spp. is commonly called tapeworms and can be transmitted to humans through the ingestion of contaminated uncooked meat. A similar study, implied that dogs that are found positive for taeniid type eggs might have obtained the infection through the type of food fed to it [21].

Statistical analyses (Table-3) revealed that age, breed, and sex did not have a significant association with the presence of parasites. These results were similar to other studies where age, sex, and breed did not play a role in the presence of parasites [8,10,23]. Some studies imply that male dogs tend to have higher parasitic egg counts [24-26]. A study reported that young dogs tend to be more susceptible to acquiring GIT parasites [26].

**Table-3:** Association of the different risk factors with the presence of parasites.

Parameter	df	Chi-square	p-value
Category (household or shelter)	1	38.07	0.00
Deworming administration	1	58.684	0.00
Contact with other dogs	5	36.022	0.00
Area where it spends most of the time	6	47.39	0.00
Playing in the grass area	2	5.999	0.05
Area where it defecates	6	16.033	0.01
Contact with other animals	3	22.539	0.00
Body score	3	38.817	0.00

On the other hand, results also showed that dogs, whether they were shelter or household, had a significant association with the presence of GIT parasites. Dogs in the shelter are expected to have higher parasite burden than those owned dogs [23]. Similarly, the body score of the dog was found as a significant factor. Body condition score is the measure of the relative body condition of an organism. The ideal body condition score in dogs is three, values that fall below three means that the dog is underweight, while values above three means that the dog is overweight [27]. One of the symptoms of a GIT infection is weight loss. A dog having a body condition score below three may imply that it is underweight due to the presence of parasites.

The rearing practices of the owners were found to be associated with the presence of parasites in their dogs. The environment where the dog stays in could potentially be a source of different parasites. A warm and moist environment will favor the growth and development of different GIT parasites. Other dogs or animals that may be infected with GIT parasites excrete their feces in the soil or grass. Dogs acquire GIT parasites from ingesting the eggs that were shed by other dogs or other animals [15].

The way the dog was reared all had a significant association with the presence of parasites (contact with other dogs, where it spends most of its time, whether or not it plays in the grass and whether or not it has contact with other dogs). Rearing can influence the exposure of the animals to the parasite source. Dogs can get intestinal parasites when it ingests the eggs shed by other dogs or other animals. These eggs are usually shed on the soil or grass [15].

The use of anthelmintics also had a significant association with the presence of parasites. A dog given a deworming treatment has a lesser chance of acquiring parasites because dewormers can rid the body of intestinal parasites. Thus, educating clients to regularly have the dogs dewormed will be very useful [28].

Proper deworming medication as treatment or prevention must be administered to dogs, especially those from the shelter homes. On the other hand, the use of molecular methods may also be performed to confirm identification and establish genetic diversity of the detected parasites in the fecal samples.

## Conclusion

GIT parasites were detected in both owned and shelter dogs in Cebu, Philippines. Of the 200 fecal samples examined, 122 samples were found to be positive with parasites. Samples from shelter dogs (90%) were found to have more parasites compared to those from owned dogs (45.4%). The most common GIT parasites detected were *Ancylostoma* spp. (38%), *Trichuris* spp. (12.5%), *Toxocara* spp. (11.5%), *Cystoisospora* spp. (8%), *Taenia* spp. (3%), and *Hammondia* spp. (1.5%). Significant associations were found between the presence of parasites and the body score, deworming status, and some rearing practices.

## Authors’ Contributions

MFMU, RHDY, and APY conceptualized the study. MFMU and RHDY contributed equally and took charge of the sample collection and laboratory analysis. APY performed the data analyses and gave valuable insights and support in the conduct of the study. All authors finally read and approved the final manuscript.
